# Diet-microbiome interactions influence lung function in chronic obstructive pulmonary disease

**DOI:** 10.3389/frmbi.2024.1426150

**Published:** 2024-10-22

**Authors:** Haowen Qiu, Rees Checketts, Mariah Kay Jackson, Jean-Jack M. Riethoven, Nadia N. Hansel, Kristina L. Bailey, Corrine Hanson, Derrick R. Samuelson

**Affiliations:** ^1^ Bioinformatics Core Research Facility, Nebraska Center for Biotechnology, University of Nebraska-Lincoln, Lincoln, NE, United States; ^2^ Department of Internal Medicine, Internal Medicine Residency Program, Creighton University Medicine Center, Omaha, NE, United States; ^3^ Department of Medical Sciences, Medical Nutrition Education Program, University of Nebraska Medical Center, Omaha, NE, United States; ^4^ Department of Medicine, Johns Hopkins Bloomberg School of Public Health, Baltimore, MD, United States; ^5^ Division of Pulmonary, Critical Care, and Sleep, Department of Internal Medicine, University of Nebraska Medical Center, Omaha, NE, United States; ^6^ Department of Veterans Affairs, Veterans Affairs (VA) Nebraska-Western Iowa, Omaha, NE, United States; ^7^ Nebraska Food for Health Center, University of Nebraska-Lincoln, Lincoln, NE, United States

**Keywords:** COPD, diet, fiber, lungs, microbiome, omega-3 fatty acid

## Abstract

Chronic Obstructive Pulmonary Disease (COPD) affects 30 million Americans. Previous epidemiologic work has shown that diet can impact pulmonary function in those with and without COPD. Diet is also a major driver of gut microbiome composition and function. Importantly, the gut microbiome has also been associated with lung health (i.e., the gut-lung axis) in both preclinical and clinical studies. Despite this growing body of evidence, many questions remain regarding the gut-lung axis. Specifically, how the microbiome impacts the relationship between diet factors and spirometry or stage of disease in people with COPD is little understood. We hypothesize that there are taxonomic differences in the gut microbiome among the different stages of COPD and that diet microbiome interactions influence pulmonary function. This study aimed to identify how the GI microbiota correlated with the severity of respiratory disease in COPD patients and how the microbiome may mediate the relationship between diet, including fiber and omega-3 fatty acids, and lung function outcomes.

## Introduction

Chronic Obstructive Pulmonary Disease (COPD) affects 30 million Americans (Prevention CfDCa, 2022) and was the sixth overall cause of death in 2021 (Prevention CfDCa, 2023). However, prior to the COVID-19 pandemic, it was the third leading cause of death worldwide (Organization WH). In addition to excess mortality, COPD increases morbidity, often leading to disability. COPD disproportionately affects women compared to men (Prevention CfDCa, 2022), as well as those who are socioeconomically disadvantaged (Buttery et al., 2021). The leading cause of COPD is tobacco smoke, with cigarette smoking being responsible for 8 out of 10 COPD-related deaths (Prevention CfDCa; Services USDoHaH, 2014). Despite smoking rates in the United States being cut in half since 1964, between 2011 and 2020, the prevalence of COPD remained unchanged. Likewise, the overall age-adjusted death rate has not changed from 1999-2019. These data emphasize smoking cessation alone may not be sufficient to optimize lung function outcomes in smokers, creating a need to examine additional modifiable lifestyle factors, such as dietary intake, as a complementary strategy for improving lung health.

Previous epidemiologic work has shown that diet can impact pulmonary function in those with COPD (Hanson et al., 2014). A “Western” diet pattern, generally categorized as high in refined grains, cured and red meats, added sugars, and fat, has also been identified as a risk factor for COPD (Young and Hopkins, 2018; Scoditti et al., 2019), impacting both men (Varraso et al., 2007b) and women (Varraso et al., 2007a). However, individual dietary factors, such as fiber and omega-3 fatty acids, have previously been identified as protective against COPD (Kan et al., 2008; Varraso et al., 2010; Hanson et al., 2016; Young et al., 2016; Saeed et al., 2020; Ding et al., 2021; Qu et al., 2022) and are potentially associated with reduced respiratory symptoms (Lemoine et al., 2019). One possible mechanism for the positive impact of fiber and omega-3 fatty acids on COPD is through supporting the gut microbiome. The gut microbiome is known to have a profound impact on the immune system, metabolism, and homeostasis. Through its metabolites, the gut microbiota impacts tissues far beyond the intestinal mucosa (Moloney et al., 2014; Albillos et al., 2020; Zhao et al., 2021; Ashique et al., 2022). Increasingly, the gut microbiome is being associated with lung health. This is becoming termed the “gut-lung axis” as an encompassing term for the cross-communication between intestinal and lung microbiota and resultant co-regulation of local and systemic response to environmental and intrinsic exposures (Ding et al., 2021; Zhao et al., 2021; Ashique et al., 2022; Qu et al., 2022). Through this association, we now know that noxious pulmonary exposures (Buttery et al., 2021), including cigarette smoke (Ding et al., 2021), can induce intestinal dysbiosis. However, dietary factors, including fiber and omega-3 fatty acids, are also key modifiers of the gut microbial composition, including microbes that produce anti-inflammatory short-chain fatty acids (So et al., 2018; Watson et al., 2018; Wagenaar et al., 2021).

Despite this growing body of evidence, many questions remain regarding the gut-lung axis. Spirometry measures the maximum volume and duration a participant can inspire and then forcefully and completely expire. These tests are considered the gold standard for the measurement of lung function, allowing for the classification of COPD severity. Little is known about whether microbiome diversity correlates with the Global Initiative for Obstructive Lung Disease (GOLD) severity stages of COPD and how nutrient intake might influence the microbiome and mediate lung function and stage of disease in people with COPD. We hypothesize that there are taxonomic differences in the gut microbiome among the different stages of COPD. This study aimed to identify the associations between nutrient intakes and respiratory disease stage in COPD patients, as well as how the microbiome may mediate the relationship between diet, including fiber and omega-3 fatty acids, and lung function outcomes.

## Materials and methods

### Patient enrollment

This study is a secondary analysis of data collected in the Comparing Urban and Rural Effects on COPD (CURE COPD, NIH grant #P50-ES026096) Center at Johns Hopkins University (JHU). The urban cohort of CURE COPD is an observational study of 99 former smokers with moderately severe COPD, followed longitudinally for six months. CURE COPD participants were evaluated at baseline, 3, and 6 months, with assessment of lung function, diet, and collection of stool samples. Inclusion criteria were 1) age ≥ 40 years, 2) physician diagnosis of COPD, 3) GOLD Stage I-IV disease with FEV_1_/FVC ≤70% and FEV_1_ (% predicted) < 80%, 4) tobacco exposure ≥ 10 pack-years, and 5) former smoker (identified those who report no current smoking in the past one year and have exhaled CO levels ≤ 6ppm. This threshold was chosen to maximize the chance of distinguishing true smokers and ex-smokers (> 95%). Exclusion criteria include; 1) use of chronic systemic corticosteroids (≥ 3 months continuous), 2) BMI less than 18.5, and 3) pregnancy or breastfeeding. The catchment area of the study represents a low socioeconomic status (SES) population from Baltimore and surrounding areas with high rates of Medicaid coverage (90%), 78% have a high school education or less, and 57% report less than $15,000 of yearly household income. All studies were approved by the JHU IRB.

### Sample collection

Participants were given a fecal sampling kit with detailed instructions. Using an established protocol, the subject is asked to swab fresh feces from toilet paper and insert the swab into a sterile screw-cap tube with cell lysis and DNA stabilization buffer (American Gut Project). The subject is then given a postage-paid mailing pouch for return to the study coordinator. Samples collected for this study were obtained prior to the COVID-19 pandemic.

### Primary outcome

The primary outcomes of the study were lung function and COPD stage. Lung function was assessed as FEV_1_ and FEV1% predicted (FEV_1_, adjusted for age, height, and sex) according to ATS guidelines (Miller et al., 2005) using a KOKO^®^ (Pulmonary Data Services, Inc., Louisville, CO) pneumotach. Predicted values for FEV_1_ were calculated by formulae of Hankinson et al. (1999). At each test, three sets of values were obtained, and the highest set of FEV_1_ and FVC measurements was used. Global Initiative for Obstructive Lung Disease (GOLD) criteria was used to classify COPD severity (Fromer and Cooper, 2008).

### 16S rRNA library preparation and sequencing

Analysis of the microbiome was performed on stool samples collected from patients. The DNA quality was checked using a Qubit 3.0 fluorometer before 16S Metagenomic Sequencing Library Preparation following Illumina MiSeq pair-end protocol. The protocol targeted the variable 16S V3 and V4 regions. After libraries were quantified and normalized, a 4nM pool of all samples was denatured and diluted to 8 pM. This pool was loaded onto the Illumina MiSeq for a 300bp paired-end run using the MiSeq v3 600 cycle kit.

### Bioinformatics

Sequences were demultiplexed using the Illumina software, according to the manufacturer’s guidelines. Bioinformatics analyses were performed following the Bioconductor workflow for microbiome data analysis by Callahan et al. (Callahan et al., 2016) using the R software (version 4.2.1). For denoising, the R package DADA2 (Callahan et al., 2016) (version 1.18.0) was used following these conditions: the forward reads were truncated at position 290 while the reverse ones were truncated at position 260 to discard positions for which nucleotide median quality was Q25 or below. High-quality sequencing reads were clustered to infer amplicon sequence variants (ASV), and a final table of ASV counts per sample was generated after removing chimeras. A naïve Bayes taxonomy classifier (Wang et al., 2007) was used to classify each ASV against the SILVA 138.1 reference database, while MAFFT (Katoh and Standley, 2013) (version 7.407) and FASTTREE (Price et al., 2009) (version 2.1.11) programs were used to construct a phylogenetic tree. Taxa abundances were normalized with the total sum scaling normalization method, dividing each ASV count by the total library size to yield their relative proportion of counts for each sample. Alpha diversity was studied with several diversity indices with the R packages phyloseq (McMurdie and Holmes, 2013) (version 1.34.0) and picante (Kembel et al., 2010) (Version 1.8.2). Principal coordinates analysis (PCoA) via various distance matrices (Bray Curtis, Jaccard, weighted and unweighted UniFrac) was used to evaluate beta diversity and to plot patterns of microbiome community diversity. Analysis of variance of the distance matrices was performed with the nonparametric MANOVA test Adonis with 999 permutations (PERMANOVA) as implemented in the R package vegan (Oksanen, 2022) (version 2.5-7).

### Dietary intake methodology

Nutrient intake was assessed using the Willett Food Frequency Questionnaire (FFQ). The FFQ provides information on the usual intake of many foods, food groups, and supplements. From responses to the questionnaire, individualized nutrient intake can be calculated based on the known nutrient content of foods. The FFQ was analyzed by trained personnel at the Harvard School of Public Health. Nutrients of interest for this analysis were based on previous associations with lung function in the literature and included total fiber intake, total omega (n)-3 fatty acid intake (FA) (including alpha-linolenic acid), intake of specific n-3 FAs [Docosahexaenoic acid (DHA) + eicosapentaenoic acid (EPA)], and total n-6 fatty acids. Using this data, a ratio of total n-6:total n-3 FAs was calculated.

### Statistics and data analysis

Prior to analysis, all data was tested for normality using the D’Agostino & Pearson test method. All data not normally distributed was log-transformed prior to analysis. Differential abundance analyses were performed using the R package corncob (Martin et al., 2020) (version 0.2.0) to reveal statistically significantly changed taxa. Regularized Canonical Correlation Analysis (rCCA) was performed using R package mixOmics (Rohart et al., 2017) (version 6.14.1) to explore associations between nutrient intake, microbiome, and pulmonary outcomes. rCCA is a standard method for microbiome studies and accounts for the high dimensionality and/or high collinearities of the datasets, both of which are commonly seen in microbiome and biological studies. For taxa differential abundance analysis, the Benjamini-Hochberg (BH) procedure (Benjamini and Hochberg, 1995) was applied to correct for multiple hypothesis testing. Mediation analyses were performed using the MODIMA method by Hamidi et al. (Hamidi et al., 2019) and the CCMM method by Sohn et al. (Michael and Hongzhe, 2019) to evaluate the mediation effect of the microbiome as a community and individual taxa after agglomerating at the genus level, respectively, between dietary covariates as exposure and lung function outcomes. Prediction of metagenomics functions was performed using PICRUSt2 (Douglas et al., 2020) (version 2.4) and Pfam (Finn et al., 2007) database of protein families. Unless specified, an adjusted p-value of less than 0.05 was used for the significance level.

## Results

### Characteristics of the study population

A total of 54 individuals who had completed fecal sample collection were included in the analysis. COPD GOLD stages were divided among the groups as follows: 7.4% (*n* = 4) GOLD stage I, 57.4% (*n* = 31) GOLD stage II, 27.8% (*n* = 15*)* stage III, and 7.4% (*n* = 4) stage IV. The mean age was 64 years, with an average BMI of 34.7. The study population was 67% female (*n* = 36) with 26 (48%) white, non-Hispanic patients and 28 (52%) black participants. Baseline characteristics of the study population are provided in **Table 1**.

**Table 1:** Baseline patient characteristics (*n* = 54).

Characteristic	COPD Stage 1 *n = 4*	COPD Stage 2 *n = 31*	COPD Stage 3 *n = 15*	COPD Stage 4 *n = 4*	Range
Mean ± SD or n (%)					
**Sex, n (% female)**	0 (0%)	21 (68%)	12 (80%)	3 (75%)	
**Age, years**	69.75 ± 11.32	63.48 ± 8.50	60.46 ± 10.78	57.75 ± 1.5	49 - 79
**Race, n (% white)**	2 (50%)	14 (45%)	7 (47%)	3 (75%)	
**BMI (kg/m^2^)**	30.44 ± 2.89	37.40 ± 9.78	36.51 ± 15.16	32.80 ± 12.91	16.4 - 64.1
**Pack-Years Smoking, pack-years**	78.5 ± 49.89	55.10 ± 38.33	39.85 ± 34.56	37.5 ± 15.00	10 - 175
**FEV_1_ (L)**	2.56 ± 0.45	1.48 ± 0.45	1.07 ± 0.22	0.88 ± 0.87	0.37 - 3.17
**FVC (L)**	3.85 ± 0.65	2.41 ± 0.64	2.12 ± 0.33	1.89 ± 0.81	1.31 - 4.63
**FEV_1_ (% Predicted)**	86.25 ± 8.09	61.87 ± 7.81	43.85 ± 5.71	30.75 ± 26.89	15 - 98
**Fiber, g**	26.92 ± 26.12	36.17 ± 36.12	22.33 ± 8.90	24.79 ± 11.56	5.48 - 166.04
**Total Omega Fatty Acids, g**	2.67 ± 4.96	0.63 ± 1.73	0.37 ± 0.30	0.20 ± 0.26	0.02 - 10.1
**Omega 3, g**	7.24 ± 11.86	3.11 ± 2.91	2.50 ± 0.85	1.47 ± 0.61	0.63 - 25
**Omega 6, g**	35.21 ± 49.70	21.40 ± 12.24	17.21 ± 7.38	11.65 ± 4.32	4.85 - 109.7
**Omega 6:3 ratio**	7.01 ± 1.76	7.90 ± 2.22	7.12 ± 1.99	8.06 ± 0.43	2.94 - 13.41

SD, Standard Deviation; BMI, Body Mass Index; FEV_1_, Forced Expiratory Volume in 1 second; FVC, Forced Vital Capacity.

### Differential microbial taxa and inferred functional capacity across COPD GOLD stages

We examined the composition of the microbiota to determine whether there were differences in the dominant phyla of bacteria of the gut microbiota between the different stages of COPD, we measured the relative abundance of the major bacterial phylum in each GOLD stage for each participant. (**Supplementary Figure 1**). In each GOLD stage, the Firmicutes and Bacteroidetes phyla dominated the gut microbial communities. Analysis of α-diversity metrics were calculated for each sample using Observed species, Chao1, and Shannon diversity measures. No significant difference in α-diversity was observed between GOLD stages (**Supplementary Figure 2A**). Similarly, analysis of β-diversity showed no significant differences in β-diversity between any GOLD stages (**Supplementary Figure 2B**).

**Figure 1:**
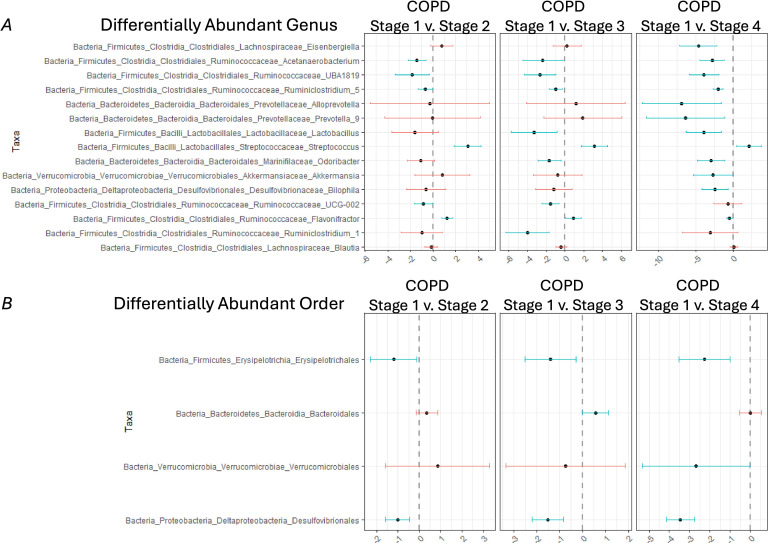
Differentially abundant taxa between different COPD Gold stages. Differentially abundant taxa at the **(A)** genus level and **(B)** order level in COPD Gold Stage 2 v 1 (left panel), COPD Gold Stage 3 v 1 (middle panel), and COPD Gold Stage 4 v 1 (right panel) using the corncob method with Benjamini-Hochberg (BH) correction for multiple comparisons. Blue lines indicate taxa that are significantly different between the compared GOLD Stages. Specifically, if the blue line is positive, then the indicated taxa is increased in GOLD Stage 2,3,4 compared to Stage 1; however, if the blue line is negative, then the indicated taxa is decreased in GOLD Stage 2,3,4 when compared to Stage 1.

We then examined the data at the genus and order level. Despite the homogeneity in the phyla, there were clear changes in the relative abundance of specific microbial taxa between GOLD stages at both the genus (**Figure 1A**) and order (**Figure 1B**) levels. In this data, the dashed lines represent the level of expression in Gold Stage 1. Data to the left of the line shows decreases, while data to the right of the line shows increases. Changes that are significant do not cross the dashed line and are in blue. Compared to GOLD stage I, there were significant decreases in the abundance of three *Ruminococcaceae* (*Acetanaerobacterium*, *UBA1819*, and *5*) (**Figure 1A**) while the abundance of *Streptococcus* increased as GOLD stage increased (**Figure 1A**). At the order level, *Erysipelotrichales* and *Desulfovibrionales* were significantly decreased in GOLD stages 2, 3, and 4 when compared to GOLD stage I (**Figure 1B**). Overall, as COPD severity increases, as measured by GOLD stage, there are more significant changes in the microbiome.

Next, we wanted to better understand the potential functional impacts of these changes in bacterial taxa that were associated with COPD GOLD stage. To do this, we performed a PICRUSt2 analysis to predict the function of the metagenome (Douglas et al., 2020). In our dataset, PICRUSt2 analysis found that there were 161 upregulated pathways (Shown in red) and 20 downregulated pathways (Shown in blue) in COPD GOLD stage 2 compared to stage 1 (**Supplementary Figure 3A**), while there were 180 upregulated pathways and 154 downregulated pathways in COPD GOLD stage 3 compared to stage 1 (**Supplementary Figure 3B**). Finally, there were 117 upregulated pathways and 308 downregulated pathways in COPD GOLD stage 4 compared to stage 1 (**Supplementary Figure 3C**). A summary of the differential pathways via PICRUSt2 and DESeq2 analysis is provided in **Supplementary Table 1**.

### Microbial taxa correlate with lung function and dietary variables

Certainly, the stage of COPD is not the only relevant clinical characteristic that could alter the microbiome. Dietary intake can also dramatically affect the microbiome. Because previous studies have shown that dietary intake can influence the rate of lung function decline, we wanted to look at dietary factors and lung function together. To do this, we performed canonical correlation analysis to evaluate the correlation between the microbiome, dietary covariates (i.e., fiber, total omega, omega-3, omega-6, and n6:n3 ratio), and lung function (i.e., FEV_1%_ predicted, FEV_1_ and FVC). A network plot and heatmap showed the strength of the association between the microbiome and these parameters (**Supplementary Figure 4**). When both lung function and dietary considerations were considered in the model, the lung function outcome FEV_1%_ predicted, and FEV_1_ were negatively associated with *Rikenella* and *Lachnospiraceae* (NK4B4 group). Likewise, FEV_1%_ predicted was also negatively associated with *Lachnospiraceae* (UCG.008), *Angelakisella*, and *Merdibacter*. Conversely, FEV_1_ and FVC were positively associated with *Desulfovibrio* (**Supplementary Figure 4A**). On the other hand, dietary parameters total omega and omega-3 were positively associated with *Succinivibrio* (**Supplementary Figure 4B**). In summary, both lung function parameters and dietary parameters are associated with significant alterations in the microbiota.

### Mediation analysis

**Figure 2:**
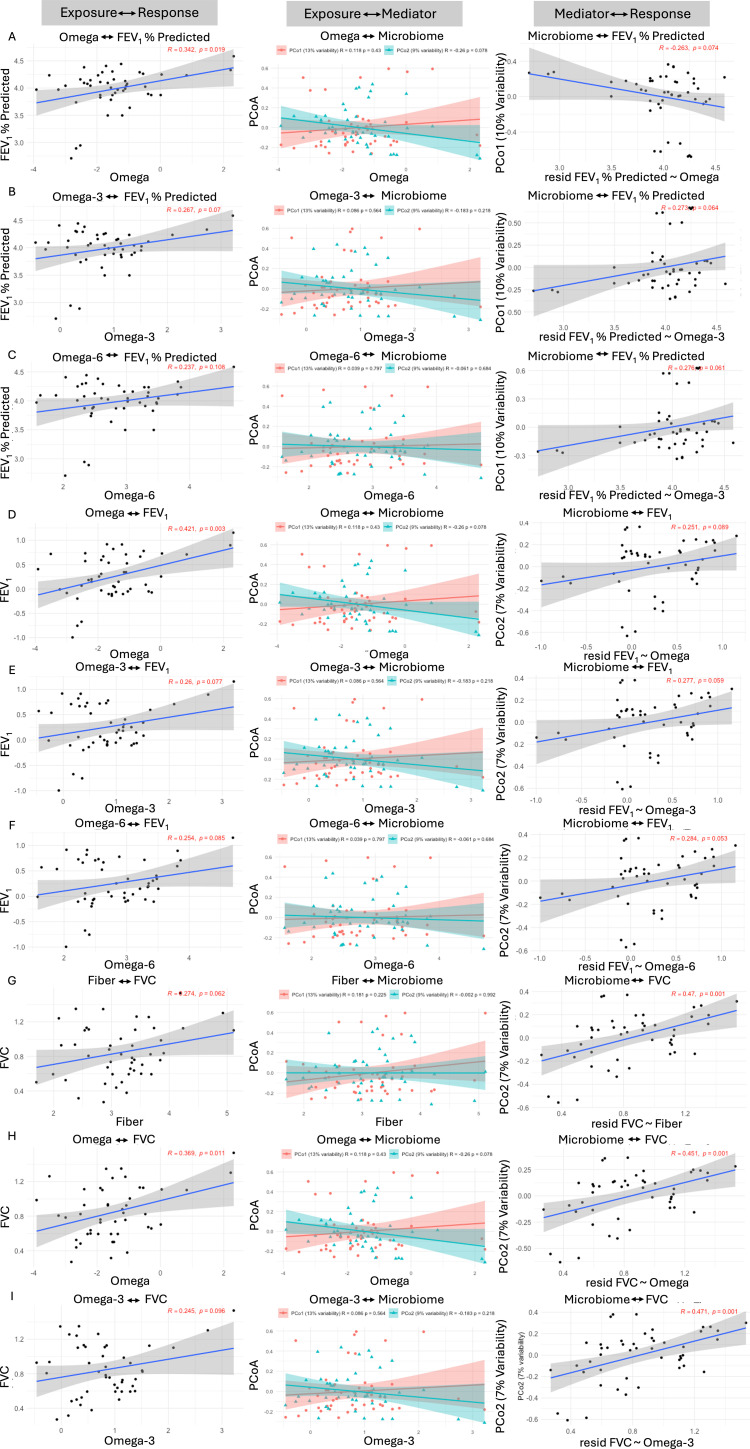
The GI microbial community partially mediates diet and lung function interactions in COPD subjects. Mediation analysis was performed with dietary parameters as the exposure and lung function parameters FEV_1_, FVC, and FEV_1_ % predicted as the outcome, and community composition as the mediator using the MODIMA method. Mediation analysis was performed using FEV1 % predicted as the outcome and **(A)** Omega, **(B)** Omega-3, and **(C)** Omega-6 as the exposures. Mediation analysis was performed using FEV1 as the outcome and **(D)** Omega, **(E)** Omega-3, and **(F)** Omega-6 as the exposures. Finally, mediation analysis was performed using FVC as the outcome and **(G)** Fiber, **(H)** Omega, and **(I)** Omega-3 as the exposures.

To understand if the relationship between diet and lung function in COPD subjects was mediated by the gut microbiota, we performed a mediation analysis. Specifically, mediation analysis was performed using dietary parameters as the exposure (fiber, total omega, omega-3, omega-6, and n6:n3 ratio) and lung function parameter (FEV_1%_ predicted, FEV_1_ and FVC) as outcome, and microbiome diversity metric Bray-Curtis as the mediator using the MODIMA method. In **Figure 2**, the first column of data shows how each dietary component influences FEV_1%_ predicted, FEV_1_, and FVC. The second column shows how each dietary component influences beta diversity (PCo1 and PCo2) of the microbiome. The third column depicts how the microbiome associated with the dietary component influences FEV_1%_ predicted, FEV_1_, and FVC. Higher levels of total omega fatty acids (**Figure 2A**), Omega-3 (**Figure 2B**), and Omega-6 fatty acids (**Figure 2C**) were associated with higher FEV_1%_ predicted. Similarly, higher levels of total omega fatty acids (**Figure 2D**), Omega-3 (**Figure 2E**), and Omega-6 fatty acids (**Figure 2F**) were associated with higher FEV_1_. Finally, levels of total fiber (**Figure 2G**), total omega fatty acids (**Figure 2H**), and Omega-3 fatty acids (**Figure 2I**) were associated with higher FVC. We identified lung function FEV_1%_ predicted, FEV_1_ and FVC, at least partially, to be significantly mediated by the microbiome when considering either fiber, total omega, omega-3, or omega-6 as exposure (**Figure 2**). However, this analysis method only provides a global test of community-level mediation, not the effects of individual components.

**Figure 3:**
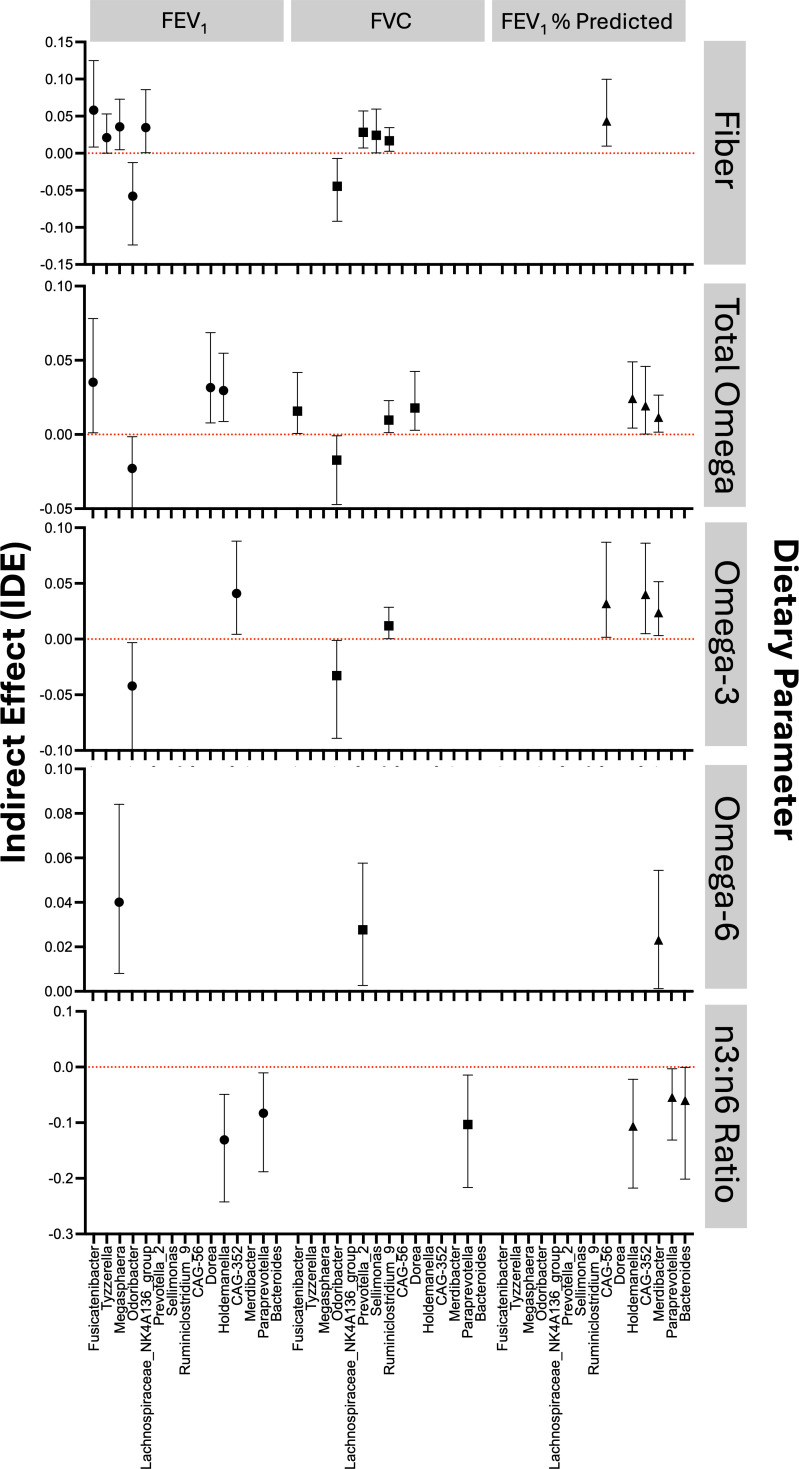
Diet and lung function interaction in COPD subjects is partially mediated via specific bacterial taxa. Mediation analysis was performed with dietary parameters as the exposure and lung function parameters FEV_1_, FVC, and FEV_1_ % predicted as the outcome, and individual bacterial taxa as the mediator using the CCMM method. Mediation analysis was performed using Fiber, total omega, omega-3, omega-6, and n6:n3 ratio (from top to bottom) as the dietary exposure parameters.

To better understand the effects of individual components of the microbiome, we performed a causal compositional mediation model (CCMM). Mediation analysis via CCMM showed indirect mediation effects of several taxa (**Figure 3**). Specifically, mediation analysis was performed using dietary parameters as the exposure (fiber, total omega fatty acid, omega-3, omega-6, and n6:n3 ratio) and lung function parameter FEV_1%_ predicted, FEV_1_ and FVC as outcomes, and individual bacterial taxa as the mediator using the CCMM method. While mediation analysis did not reveal a significant direct effect (DE) or total indirect effect (TIDE) between diet (fiber, total omega, omega-3, omega-6, and the ratio of omega-6 to omega-3), the microbiome, and FEV_1%_ predicted, FEV_1_ and FVC significant indirect component-wise mediation effects were observed. Specifically, for fiber, indirect component-wise mediation effects were seen for one bacterial taxon, *CAG-56* (IDE of 0.044 with a CI of 0.009, 0.099), on FEV_1%_ predicted. For total omega fatty acids, indirect component-wise mediation effects were seen for three bacterial taxa, *Holdemanella* (IDE of 0.024 with a CI of 0.004, 0.049), *CAG-352* (IDE of 0.019 with a CI of 0.0003, 0.045), and *Merdibacter* (IDE of 0.012 with a CI of 0.002, 0.027), on FEV_1%_ predicted. For omega-3 fatty acids, indirect component-wise mediation effects were seen for three bacterial taxa, *CAG-56* (IDE of 0.032 with a CI of 0.002, 0.087), *CAG-352* (IDE of 0.040 with a CI of 0.005, 0.086), and *Merdibacter* (IDE of 0.024 with a CI of 0.003, 0.052), on FEV_1%_ predicted. For omega-6 fatty acids, indirect component-wise mediation effects were seen for one bacterial taxon, *Merdibacter* (IDE of 0.023 with a CI of 0.001, 0.054), on FEV_1%_ predicted. For the ratio of omega-3 to omega-6 fatty acids, indirect component-wise mediation effects were seen for three bacterial taxa, *Holdemanella* (IDE of -0.106 with a CI of -0.217, -0.022), *Paraprevotella* (IDE of -0.054 with a CI of -0.131, -0.003), and *Bacteroides* (IDE of -0.060 with a CI of -0.201, -0.001), on FEV_1%_ predicted. Similar results were seen for each dietary parameter, with either FEV_1_ or FVC as the outcome variable (**Figure 3**). A summary of the mediation analysis is shown in **Supplementary Table 2**.

## Discussion

COPD is a chronic lung disease principally caused by tobacco smoking. It is characterized by airway inflammation, obstruction on pulmonary function tests, chronic cough, and phlegm production. Because it is a lung disease, most microbiome studies have focused on the flora of the lungs (Leung et al., 2017; Dicker et al., 2021; Madapoosi et al., 2022) through sampling of bronchoalveolar lavage fluid, lung tissue, or sputum. These studies have firmly established that individuals who suffer from COPD have an altered lung microbiome compared to healthy individuals. While the lung microbiome is known to contribute to COPD progression (Qu et al., 2022), the function of the gut microbiome remains understudied. In this analysis, we sought to better understand how the gut microbiome changes in the different stages of COPD. Importantly, we also wanted to understand how dietary intake of key factors such as fiber and omega 3 and 6 fatty acids influence the gut microbiome in COPD.

We began our analysis by determining whether there were changes in the gut microbiome at the phylum level between the different stages of COPD (**Supplementary Figure 1**). While we did not identify dramatic changes between stages of COPD at this very high level, our data compares favorably to previous studies showing that Firmicutes and Bacteroidetes phyla dominated the gut microbial communities in those with COPD (Trompette et al., 2014; Chiu et al., 2021; Dicker et al., 2021). Likewise, other studies have not identified changes in gut microbiota in different stages of COPD (Qu et al., 2022). We went on to look at alpha diversity (**Supplementary Figure 2A**) and beta diversity (**Supplementary Figure 2B**) at the phylum level between the stages of COPD. We did not measure any changes at this level. This is similar to previously published studies (Qu et al., 2022). This indicates that our methods and patient selection compare favorably to those already published.

Despite the lack of changes at the phylum level, we did measure changes in the gut microbiome in both the order (**Figure 1B**) and Genus level (**Figure 1A**) with increasing COPD stage. One of the most consistent changes was increased *Streptococcus* species in stages II, III, and IV compared to stage I (**Figure 1A**). This data is consistent with previous reports of increases in *Streptococcus* species in the gut microbiome of COPD patients compared to control (Bowerman et al., 2020). Bowerman et al. report increased abundances of both *Streptococcus sp000187445* and *Streptococcus vestibularis*. In that study, these strains were correlated with reduced lung function (Bowerman et al., 2020). Likewise, in a study of acute exacerbations of COPD, researchers found an increased abundance of both *Streptococcus parasanguinis_B* and *Streptococcus salivarius* in the fecal microbiome (Chan et al., 2015). Interestingly, streptococcus species are also increased in the upper GI tract microbiome of current smokers compared to never smokers (So et al., 2018).

We went on to examine what effect the differing microbiome could have. Compared to the microbiome of stage I COPD, Stages II, III, and IV had multiple proteins that are predicted to be differentially expressed. Many of these genes were upregulated up to nearly 30-fold, suggesting that this could make a dramatic difference in the microenvironment. This suggests that the changes in the composition of the microbiota have the potential to create physiologic differences. We observed changes in predicted functional protein families for lipid, xenobiotic, and amino acid metabolism. Like previous studies, we found significant correlations between lung function (FEV_1%_ predicted, FEV_1_ and FVC) and the intestinal microbiota; FEV_1%_ predicted and FEV_1_ were negatively associated *Rikenella* and *Lachnospiraceae* (NK4B4 group). Likewise, FEV_1%_ predicted was also negatively associated *Lachnospiraceae* (UCG.008), *Angelakisella*, and *Merdibacter*. Conversely, FEV_1_ and FVC were positively associated with *Desulfovibrio*.

Diet is also an important factor influencing both COPD and the composition of the intestinal microbiota (Sorli-Aguilar et al., 2016; Jang et al., 2020; Lai et al., 2022; Kawashima et al., 2024). Dietary fiber content changes the composition of the GI microbiota by altering the ratio of *Firmicutes* to *Bacteroidetes* (Trompette et al., 2014; Chiu et al., 2021; Dicker et al., 2021), which can directly affect how the gut microbiota metabolizes fiber. The metabolism of fiber by the GI microbiota results in the production of short-chain fatty acids (SCFA), which have been shown to have anti-inflammatory properties. Intestinal microbiota-mediated production of various SCFAs is important for host systemic immunity (Meijer et al., 2010; den Besten et al., 2013; Trompette et al., 2014). SCFAs aid in the control of both asthma and allergic inflammation in the lungs (Trompette et al., 2014; Saeed et al., 2020). For example, mice fed a high-fiber diet had increased circulating levels of SCFAs and were protected against allergic inflammation in the lung, whereas a low-fiber diet decreased levels of SCFAs and increased allergic airway disease (Saeed et al., 2020). In addition, recently in mouse models of cigarette smoke induced COPD, it was found that fecal microbiota transfer of “healthy” microbiota mitigated inflammation, alveolar destruction, and impaired lung function associated with COPD. Furthermore, COPD markers correlated with the relative abundance of *Muribaculaceae, Desulfovibrionaceae* and *Lachnospiraceae* family members. Finally, glucose and starch metabolism were significantly downregulated in the COPD-associated microbiota, and supplementation of mice or human patients with complex carbohydrates improved disease outcomes (Budden et al., 2024).

With regards to fatty acids, recent evidence has highlighted the fact that these compounds have the potential to remodel the gut microbiome, and that these changes may modulate lung metabolism (Li et al., 2023). While these findings have been mostly in animal experiments, our study provides important confirmation that the gut-lung axis can be modified by nutrition intake. Using two different mediation analyses, we found that the relationship between diet and lung function was partially mediated by the intestinal microbiota, at both a community level and individual taxa level. Specifically, fiber effects on FEV_1%_ predicted via the gut microbiome were driven primarily by 1 bacterial taxon (e.g., *CAG-56)*. Fiber effects on FEV_1_ via the gut microbiome were driven by five bacterial taxa (e.g., *Fusicatenibacter*). Likewise, fiber’s effects on FVC via the GI microbiome were driven by four bacterial taxa (e.g., *Prevotella_2*). In other words, our data suggest that as dietary fiber increases the abundance of *CAG-56, Fusicatenibacter*, *Megasphaera*, and *Lachnospiraceae_NK4A136_group* increase while decreasing the abundance of *Odoribacter*, which together improve lung function (FEV_1%_ predicted, FEV_1_, and FVC). Similar results were observed when evaluating the relationship between total dietary omega-fatty acids and lung function, which was partially mediated by the intestinal microbiota at both the community level and individual taxa level. Total dietary omega-fatty acids effects on FEV_1%_ predicted via the GI microbiome were driven by 3 bacterial taxa, *Holdemanella, CAG-352*, and *Merdibacter.* Total dietary omega-fatty acids effects on FEV_1_ were driven by four bacterial taxa (e.g., *Fusicatenibacter*). Likewise, total dietary omega-fatty acids effects on FVC via the gut microbiome were driven by two bacterial taxa (e.g., *Odoribacter*). In other words, as total dietary omega-fatty acids increase the abundance of *Fusicatenibacter*, *Holdemanella*, and *Dorea* increase while decreasing the abundance of *Odoribacter*, which together leads to improved lung function (FEV_1%_ predicted, FEV_1_, and FVC). These results are supported by previously published data as *Fusicatenibacter*, *Megasphaera*, *Lachnospiraceae_NK4A136_group, Holdemanella*, and *Dorea* have all previously been shown to increase with the addition of dietary fiber (Burns et al., 2018; Myhrstad et al., 2020; Burakova et al., 2022; Huang et al., 2023; Zhao et al., 2024). Interestingly, the reduction in *Odoribacter* and associated improvements in lung function is in line with similar studies. For example, Nontuberculous mycobacterial infected mice showed decreased levels of L-arginine in sera, and oral administration of L-arginine or fecal microbiota transplantation from supplemented mice mitigated NTM infection and increased macrophage and Th1 responses. These changes were also associated with alteration to the gut microbiota, with an increased relative abundance of *Bifidobacterium*, *Bilophila*, and unclassified YS2 and a decreased relative abundance of *Odoribacter*, *Prevotella*, and *Akkermansia* (Kim et al., 2022). Similarly, in another study, *Odoribacter* was identified to have the strongest impact on the development of squamous cell lung carcinoma (Chen et al., 2024). Conversely, increases in *Fusicatenibacter* and associated improvements in lung function are similar to studies that identified the changes in the relative abundance of specific genera, which were associated with significant alterations in fecal metabolites of patients with tuberculosis (TB) (Wang et al., 2022). Specifically, *Bacteroides*, *Parabacteroides*, *Fusobacterium*, and *Lachnoclostridium* were enriched in TB patients while *Blautia*, *Roseburia*, *Bifidobacterium*, *unidentified Ruminococcaceae*, *Fusicatenibacter*, and *Romboutsia* were enriched in healthy people, all of which was associated with a decrease the production of SCFAs (Wang et al., 2022). However, additional experiments need to be performed to validate a functional mediation effect of the microbiota in relationship to all dietary and lung functional parameters. While our study focused on critical dietary exposures with biological plausibility of impacting the gut microbiome and lung function, future exploration of additional dietary components, such as more pro-inflammatory foods, and other potential confounders, such as sex, age, and race should be included in larger studies with adequate analysis power. In conclusion, our study strengthens established associations between the colonization of *Streptococcus* and COPD (Leung et al., 2017; Bowerman et al., 2020; Dicker et al., 2021; Madapoosi et al., 2022; Sim et al., 2022), as well as provides novel insight into the relationship between diet, the microbiome, and lung function in subjects with COPD.

## Data Availability

The datasets presented in this study can be found in online repositories. The names of the repository/repositories and accession number(s) can be found in the article/**Supplementary Material**.
