# Supplementary material for: Diet-microbiome interactions influence lung function in chronic obstructive pulmonary disease
Source: Front Microbiomes. 2024 Oct 22;3:1426150. doi: 10.3389/frmbi.2024.1426150 (PMC12094515; doi:10.3389/frmbi.2024.1426150)
Supplement: Supplementary file 1 [file DataSheet1.docx]

Supplementary Material

# SUPPLEMENTAL DATA

# Sequencing data is deposited in the National Center for Biotechnology Information Sequence Read Archive (BioProject ID: PRJNA1031142).

# SUPPLEMENTAL TABLES

**Supplemental Table 1**. Differentially abundant inferred protein families between different COPD Gold stages.

**Supplemental Table 2**. A summary of the mediation analysis causal direct effect and total indirect effect via ccmm.

# SUPPLEMENTAL FIGURE LEGENDS

**Supplemental Figure 1. Relative abundance of the major phylum in different COPD Gold stages.** Relative abundance of microbial taxa in human fecal samples by COPD GOLD stage. Taxa abundances were normalized with the total sum scaling normalization method.


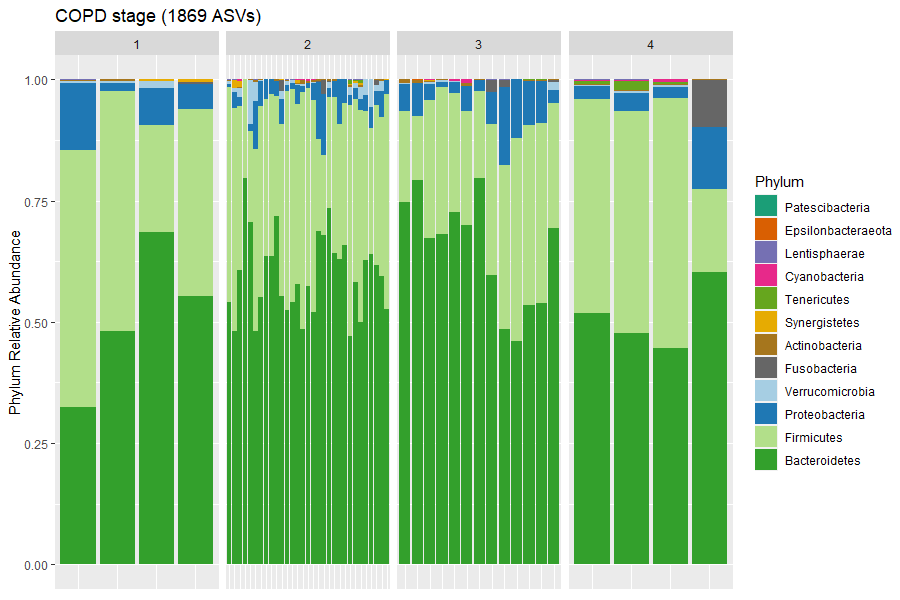


**Supplemental Figure 2. Alpha and beta diversities from different COPD Gold stages.** (A) Alpha diversity metrics Observed species, Chao1, and Shannon diversity were Calculated. (B) Beta diversity was calculated using Bray-Curtis distance, Jaccard distance, as well as weight and unweighted Unifrac distances. There was no significant difference in any alpha diversity or beta diversity metric, as determined by pairwise Wilcoxon rank sum tests and PERMANOVA, respectively.


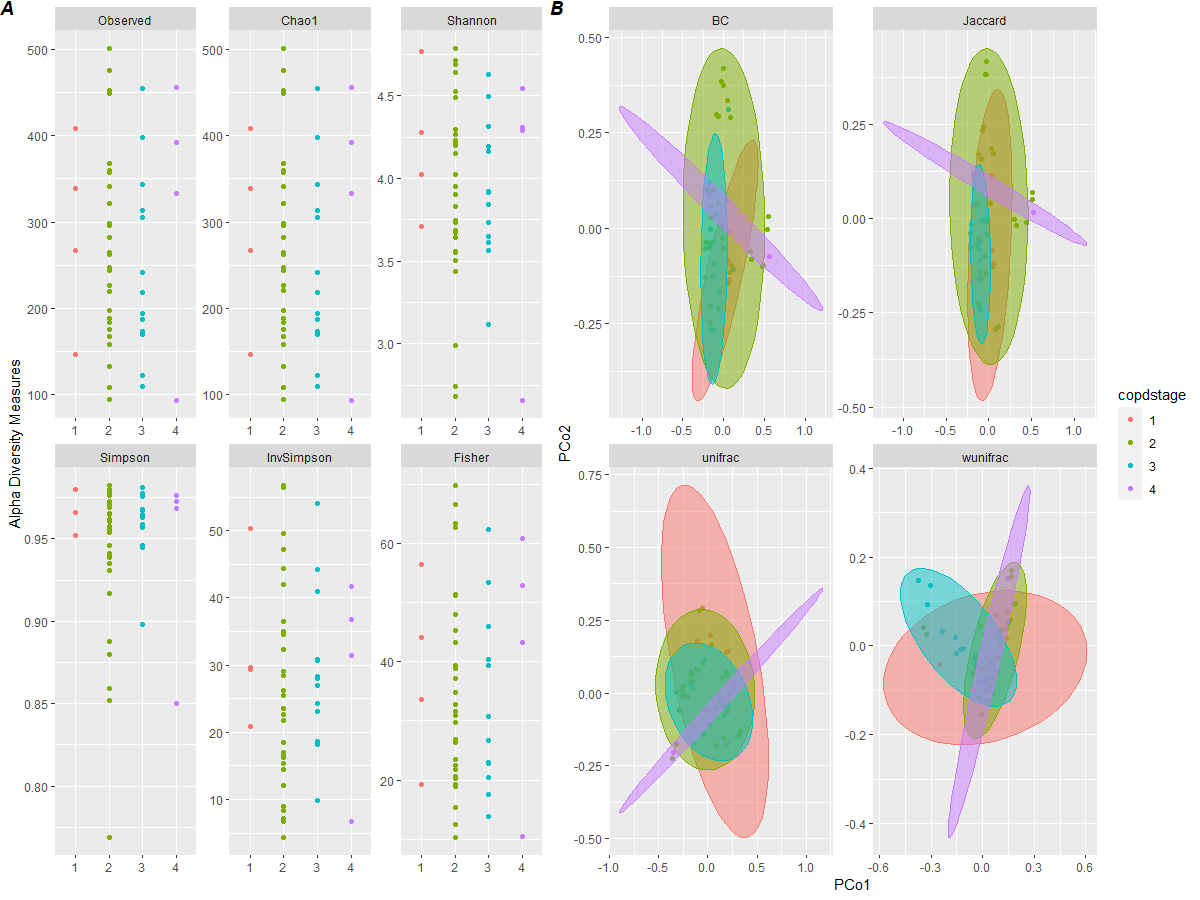


**Supplemental Figure 3. Differentially abundant inferred protein families between different COPD Gold stage.** Differentially abundant protein families between (A) COPD Gold Stage 2 v 1, (B) COPD Gold Stage 3 v 1, and (C) COPD Gold Stage 4 v 1. Red dots are significantly upregulated (log2FC > 1) in stage 2, 3, or 4 compared to stage 1, while blue dots are significantly downregulated (log2FC < 1) in stage 2, 3, or 4 compared to stage 1. Labels added for the top 10 upregulated and downregulated protein families.

GOLD II GOLD III GOLD IV


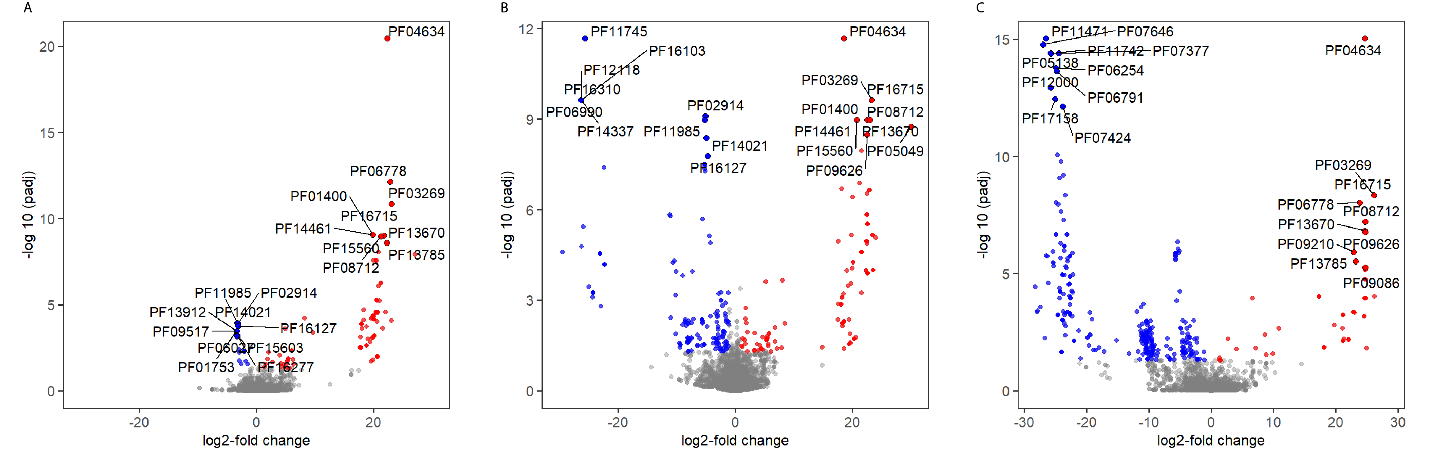


**Supplemental Figure 4. Canonical correlation analysis of the microbiome with lung and dietary parameters.** (A) Network plot (top) and heatmap (bottom) of the lung function parameters and associated organisms. (B) Network plot (top) and heatmap (bottom) of the dietary parameters and associated organisms.
